# Application of Intravoxel Incoherent Motion Diffusion-Weighted Imaging in Predicting and Monitoring Early Efficacy of Anti-Angiogenic Therapy in the C6 Glioma Rat Model

**DOI:** 10.3389/fonc.2021.842169

**Published:** 2022-01-28

**Authors:** Weishu Hou, Yangyang Xue, Yinfeng Qian, Hongli Pan, Man Xu, Yujun Shen, Xiaohu Li, Yongqiang Yu

**Affiliations:** ^1^ Department of Radiology, First Affiliated Hospital of Anhui Medical University, Hefei, China; ^2^ Department of Nuclear Medicine, The First Affiliated Hospital of Anhui Medical University, Hefei, China; ^3^ Department of Basic Medical Sciences, School of Basic Medical Sciences, Anhui Medical University, Hefei, China

**Keywords:** intravoxel incoherent motion diffusion-weighted imaging (IVIM-DWI), glioma, angiogenesis inhibitors, therapy, vascular normalization

## Abstract

**Objective:**

To investigate the feasibility of intravoxel incoherent motion (IVIM) diffusion-weighted imaging (DWI) in evaluating early effects of anti-angiogenic therapy in the C6 glioma rat model.

**Methods:**

Twenty-six rats of the C6 glioma model were randomly divided into a treatment group (received bevacizumab) and a control group (physiological saline). IVIM-DWI was performed on days 0, 1, 3, 5, and 7 after anti-angiogenic therapy and tumor growth and IVIM-DWI parameters were dynamically observed. Hematoxylin and eosin, CD34 microvessel density (MVD), proliferation of cell nuclear antigen (PCNA), and Hif-α staining were performed on day 7. One-way ANOVA was used to compare intra-group differences and an independent-samples *t*-test was used to compare inter-group differences of MRI parameters. Correlations between IVIM-DWI parameters, tumor size, and pathological results were analyzed.

**Results:**

The relative change in tumor volume (ΔVolume) in the two groups differed significantly on days 5 and 7 (*p* = 0.038 and *p* < 0.001). The perfusion-related parameters D*- and f-values decreased in the treatment group and demonstrated significant differences compared with the control group on days 3, 5, and 7 (*p* = 0.033, *p* < 0.001, and *p* < 0.001, respectively). The diffusion-related parameters ADC and D-values increased in the treatment group and were found to be significantly differently different from the control group on days 5 and 7 (both *p* < 0.001). The initial D-value showed a negative correlation with ΔVolume (γ = −0.744, *p* < 0.001), whereas the initial D*-value and relative change of D-value had a positive correlation with ΔVolume (γ = 0.718, *p* < 0.001 and γ = 0.800, *p* < 0.001, respectively). MVD was strongly positively correlated with D*-value (*r* = 0.886, *p* = 0.019), PCNA was negatively correlated with ADC- and D-values (*r* = −0.848, *p* = 0.033; and *r* = −0.928 *p* = 0.008, respectively), and Hif-1α was strongly negatively correlated with D*-value (*r* = −0.879, *p* = 0.010).

**Conclusion:**

IVIM-DWI was sensitive and accurate in predicting and monitoring the effects of early anti-angiogenesis therapy in a C6 glioma rat model.

## Introduction

High-grade glioma is the most aggressive primary malignant tumor of the central nervous system in adults and has a high expression of vascular endothelial growth factor (VEGF) (1). VEGF plays an essential role in regulating angiogenesis of glioma and, thus, anti-angiogenesis therapy targeting the VEGF signaling pathway has been recognized as an effective targeted therapy method for malignant glioma (2, 3). Recently the recombinant humanized monoclonal antibody bevacizumab (Avastin^®^, Bev), an anti-angiogenesis medicine, has been widely used as a treatment, either alone or in combination with traditional chemotherapy, for recurrent glioblastoma and newly diagnosed high-grade glioma (4–6).

However, to date, how to monitor the effects of anti-angiogenesis therapy sensitively, dynamically, and non-invasively is still a major challenge of glioma therapy. Diffusion-weighted imaging (DWI) is a functional magnetic resonance imaging (MRI) sequence used to evaluate or predict treatment outcome of malignant tumors; however, the apparent diffusion coefficient (ADC) has been shown to be influenced by both the diffusion of water molecules and microcirculation due to the mono-exponential model of DWI (7, 8). Recently, an intravoxel incoherent motion DWI (IVIM-DWI) has been developed that can separate microcirculation from restricted Brownian self-diffusion, and has been applied to evaluate the microcirculation of tumors after anti-angiogenic therapy several types of animal models (9–11). However, few studies have focused on the early and dynamic changes of IVIM-DWI parameters during therapy and the correlation between IVIM-DWI parameters and histological assessment.

Therefore, the purpose of this study was to observe the early dynamics of IVIM-DWI parameters during anti-angiogenic therapy to explore the accuracy of IVIM-DWI in anti-angiogenic therapy outcome *via* determining the angiogenesis, cell proliferation, and hypoxia of cells in the C6 glioma rat model.

## Materials and Methods

### Ethics Statement

All treated procedures were approved by the Ethics Committee for Animal Experimentation of Anhui Medical University of Anhui Province, China (Approval no. SCXK-Wan-2017–001) and were conducted in strict accordance with the Guidelines of the National Institutes of Health for the Care and Use of Laboratory Animals.

### Cell Culture and Animal Model Establishment

C6 glioma cells were purchased from the Institute of Biochemistry and Cell Biology (Shanghai, PR, China). The cells were kept in the incubator at 37°C in 5% carbon dioxide, and cultured in high glucose Dulbecco’s Modified Eagle’s Medium (Abcam, Cambridge, UK). After C6 cells had reached approximately 90% confluency, they were digested with 0.05% trypsin and washed twice with phosphate buffered saline (Abcam, Cambridge, UK). Finally, the C6 cell suspension, at a concentration of 1 × 10^7^ cells/ml, was prepared to establish the glioma orthotopic rat model.

Male Sprague–Dawley rats (purchased from Animal Center of Anhui Province, No. SCXK-Wan-2017–001) aged 6–8 weeks were anesthetized by intraperitoneal injection with 10% chloral hydrate (0.3 ml/100 g), and the glioma orthotopic rat model was established using a stereotactic apparatus (Kopf, Cayunga, CA, USA). The heads of the rats were shaved, disinfected with 0.1% povidone-iodine, and fixed on the stereotactic apparatus. An HP-4 dental drill bit was used to drill a hole in the skull at the following three coordinates: 1 mm to the anterior arcuate suture, 3 mm to the right of the sagittalis suture, and at a depth of 5 mm. Finally, 10 μl of the C6 cell suspension was injected into the caudate nucleus with a 0.5-ml microsyringe at a rate of 1 μl/min.

### Therapy of the Glioma Model

Rat models that were established successfully were randomly divided into a treatment group and control group. Rats in the treatment group were administered with Bev (Roche, Shanghai, China) diluted with 0.9% physiological saline, while rats in the control group were administered with vehicle (0.9% physiological saline). The two groups received intraperitoneal injection of Bev or vehicle, respectively, at a dose of 5 mg/kg once daily for 7 days.

### MRI Scanning

Prior to MRI, all rats were anesthetized with 3% isoflurane and continuous anesthesia with 1%–2% isoflurane using an anesthesia machine for small animals (Suzhou Zhongzhi Medical Technologies, China), and their core temperature maintained at 37°C. MRI scans were then performed using a 3.0-T MRI system (Discovery 750w, GE Medical System, Milwaukee, WI, USA) with a custom-built eight-channel receiver coil for animals (GE Medical System). Both the treated and control groups underwent an initial MRI scan before treatment (day 0) and then subsequent MRI sequences on days 1, 3, 5 and 7 after treatment with Bev or vehicle. MRI sequences included axial fast spin-echo T1-weighted imaging (T2W1) with the following parameters: repetition time/echo time (TR/TE) = 400 ms/9.5 ms; slice thickness = 2.0 mm; field of view (FOV) = 60 mm × 48 mm; and number of excitations (NEX) = 4.0. For axial spin echo T2-weighted imaging, the parameters included the following: TR/TE = 2,000 ms/46 ms; slice thickness = 2.0 mm; FOV = 60 mm × 48 mm; and NEX = 3.0. For IVIM DWI scans, single-shot echo-planar imaging DWI sequence was performed with nine b-values of 0, 10, 50, 100, 200, 400, 600, 800, and 1000 s/mm^2^. Other parameters included TR/TE = 3000 ms/102.4 ms; flip angle, 90°; matrix = 64 × 64; FOV = 90 mm × 90 mm; section thickness = 2.0 mm; and NEX= 4.0, with a total scanning time of 6 min and 15 s.

### Imaging Analysis

All MRI data were transferred to a post-processing workstation AW4.6 (GE Healthcare, USA), and tumor volume and IVIM-DWI parameter measurements were performed independently by one radiologist with 12 years’ experience in MRI diagnosis.

For the calculation of the glioma volume, the region of interest (ROI) was drawn along the edge of tumor on T2WI images to obtain the surface area of the tumor in each slice (mm^2^), after which the surface area of all the slices was summed and multiplied by the slice thickness (2 mm) to determine the total tumor volume (mm^3^).

For the quantitative measurement of IVIM-DWI parameters, MRI data were analyzed using MADC software (GE Healthcare, USA) in the AW4.6 post-processing workstation. First, the IVIM-DWI parameter (apparent diffusion coefficient, ADC; diffusion coefficient, D; pseudodiffusion coefficient, D*; and perfusion fraction, f) maps were generated automatically using MADC software. Next, due to the low signal-to-noise ratio of IVIM-DWI image, the ROI was set in the section that demonstrated the largest tumor diameter according to both axial T2WI image and DWI image with a *b*-value of 1000 s/mm^2^. The ROI (12–20 mm^3^) was drawn manually along the edge of the tumor in order to cover as much of the solid part as possible and exclude necrotic and hemorrhagic areas. Finally, the average values of IVIM-DWI quantitative parameters were calculated automatically.

### Histological Assessment

After the last MRI scan on the 7th day of treatment, all rats were sacrificed by cervical dislocation. Then, the brain tissue was removed, fixed with 4% paraformaldehyde, dehydrated with 70% alcohol, embedded with paraffin, and dissected into axial sections. To ensure that the tumor sections corresponded to IVIM-DWI images as accurately as possible, sections were processed sequentially at a thickness of 4 μm, in the same orientation as the axial MRI plane. Finally, hematoxylin and eosin staining was performed according to standard procedures.

To evaluate neoangiogenesis and cell proliferation of hypoxic glioma cells, sections were further analyzed by immunohistochemical staining using CD34 (1:250; Abcam), PCNA (PCNA, 1:100; Santa Cruz Biotechnology, CA, USA), and Hif1-α (1:100; Abcam) monoclonal antibodies. A pathologist with 10 years of experience reviewed the microvessel density (MVD) of CD34-positive cells and PCNA and Hif1-α expression. MVD calculation was determined by identifying three “hotspots” with dense positive cells in a low-power field (40×) and the number of CD34-positive cells was counted manually under a high-power field (200×); MVD was then calculated as the average number of the three “hotspots”. Expression of PCNA and Hif1-α was assessed using ImageJ software (National Institutes of Health).

### Statistical Analysis

All statistical processes were performed by SPSS 19.0 (IBM, Armonk, NY, USA). The tumor volume and quantitative IVIM-DWI parameters were expressed as the mean ± standard deviation. A one-way analysis of variance (ANOVA) with post-hoc test (Bonferroni test) was used to compare IVIM-DWI parameters among the five time points in the treatment and control group. An independent-samples *t*-test was used to compare IVIM-DWI parameters between the treatment and control groups. Pearson’s analysis was used to analyze the correlation between IVIM-DWI parameters and the average tumor growth rate. Pearson’s or Spearman’s correlation tests were used to analyze the correlation between IVIM-DWI parameters and histological assessment in the treatment group. *p* < 0.05 indicated a significant difference.

## Results

### Effect of Bev on Tumor Volume

**Figure 1:**
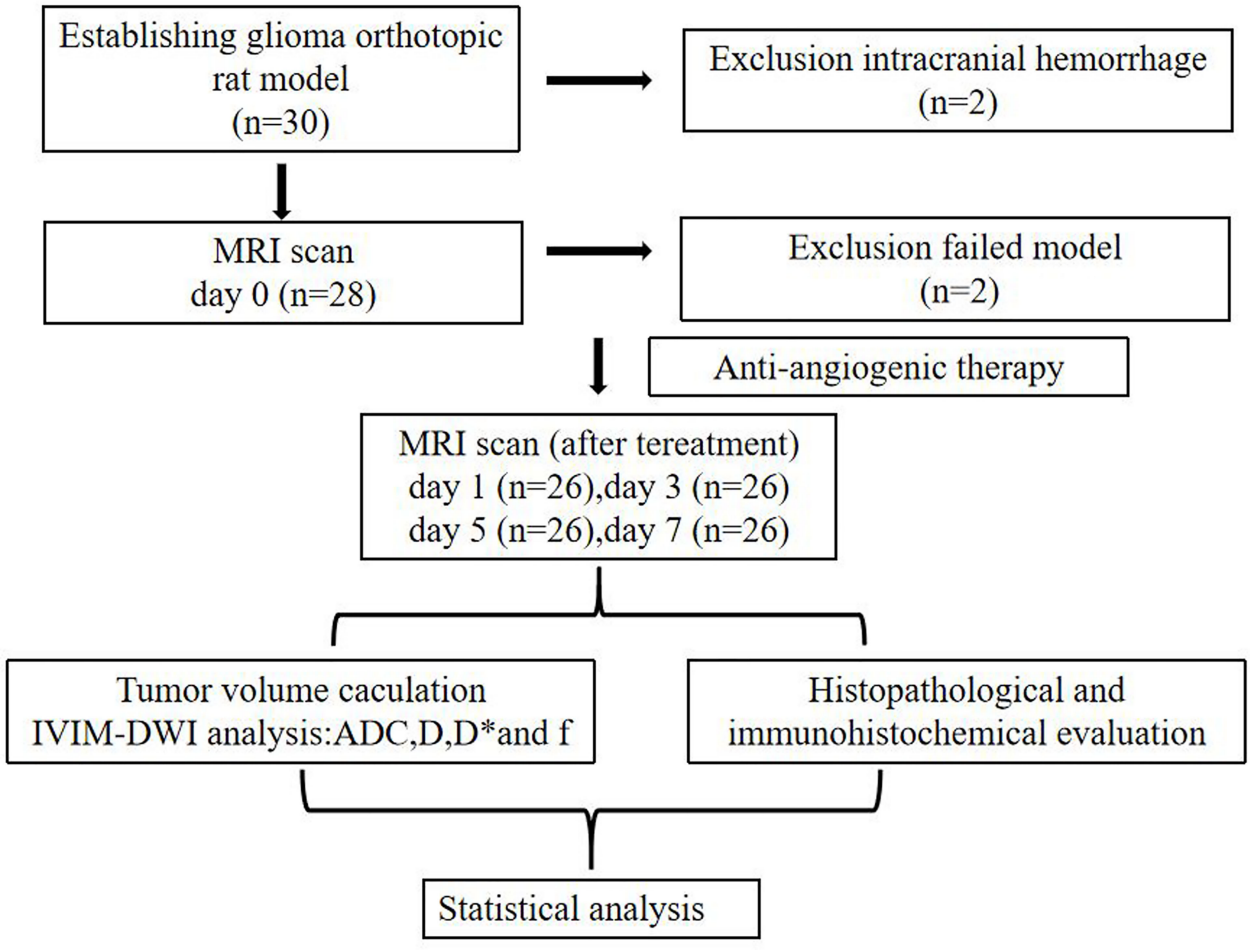
The experiment flow chart of glioma model establishment, anti-angiogenic therapy, and MRI scan. ADC, apparent diffusion coefficient; D, diffusion coefficient; D*, pseudodiffusion coefficient; f, perfusion fraction.

**Figure 2:**
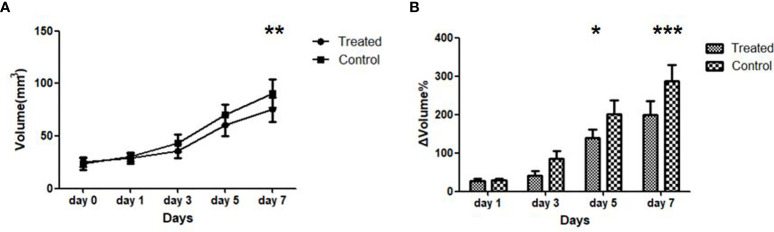
Dynamic of tumor volume size **(A)** and average tumor growth rate ΔVolume **(B)** of the treated and control group during anti-angiogenic course. *p < 0.05, **p < 0.01, ***p < 0.001. Error bars denote standard errors.

Overall, 26 out of 30 rats underwent a successful glioma orthotopic procedure; two rats died due to intracranial hemorrhage, and no tumors were found in an additional two rats. (**Figure 1**). There was no bleeding, weight loss, or other serious side effects during the treatment course of Bev or vehicle in our study. The tumor volume of the control and treatment groups before treatment (day 0) was 23.4 ± 5.58 mm^3^ and 25.2 ± 4.33 mm^3^, respectively, indicating no significant difference between the two groups (*p* = 0.76). After treatment with Bev, the average tumor volume of the two groups differed significantly on day 7 (90.7 ± 13.4 mm^3^ vs. 75.3 ± 11.5 mm^3^, *p* = 0.022, **Figure 2A**). The relative change in tumor size was calculated as ΔVolume = (volume_n_ − volume_0_)/volume_0_ × 100%. The ΔVolume in the control group was 29.1% ± 5.4% on day 1, 85.1% ± 13.5% on day 3, 200.5% ± 45.4% on day 5, and 287.6% ± 58.4% on day 7, while that of the treatment group was 27.7% ± 4.7% on day 1, 42.1% ± 12.3% on day 3, 138.8% ± 25.7% on day 5, and 198.8% ± 42.3% on day 7. The ΔVolume in the two groups differed significantly on days 5 and day 7 (*p* = 0.038 and *p* < 0.001, respectively, (**Figure 2B**).

### IVIM-DWI Parameter Images

**Figure 3:**
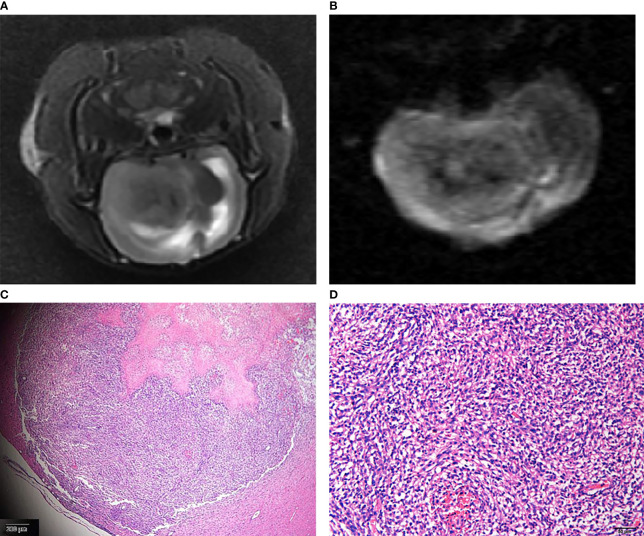
The glioma on T2WI **(A)** and IVIM-DWI (b = 1,000 s/mm^2^) **(B)** images. Corresponding HE staining showed atypical tumor cells (**C** original magnification ×4; **D** original magnification ×10).

**Figure 4:**
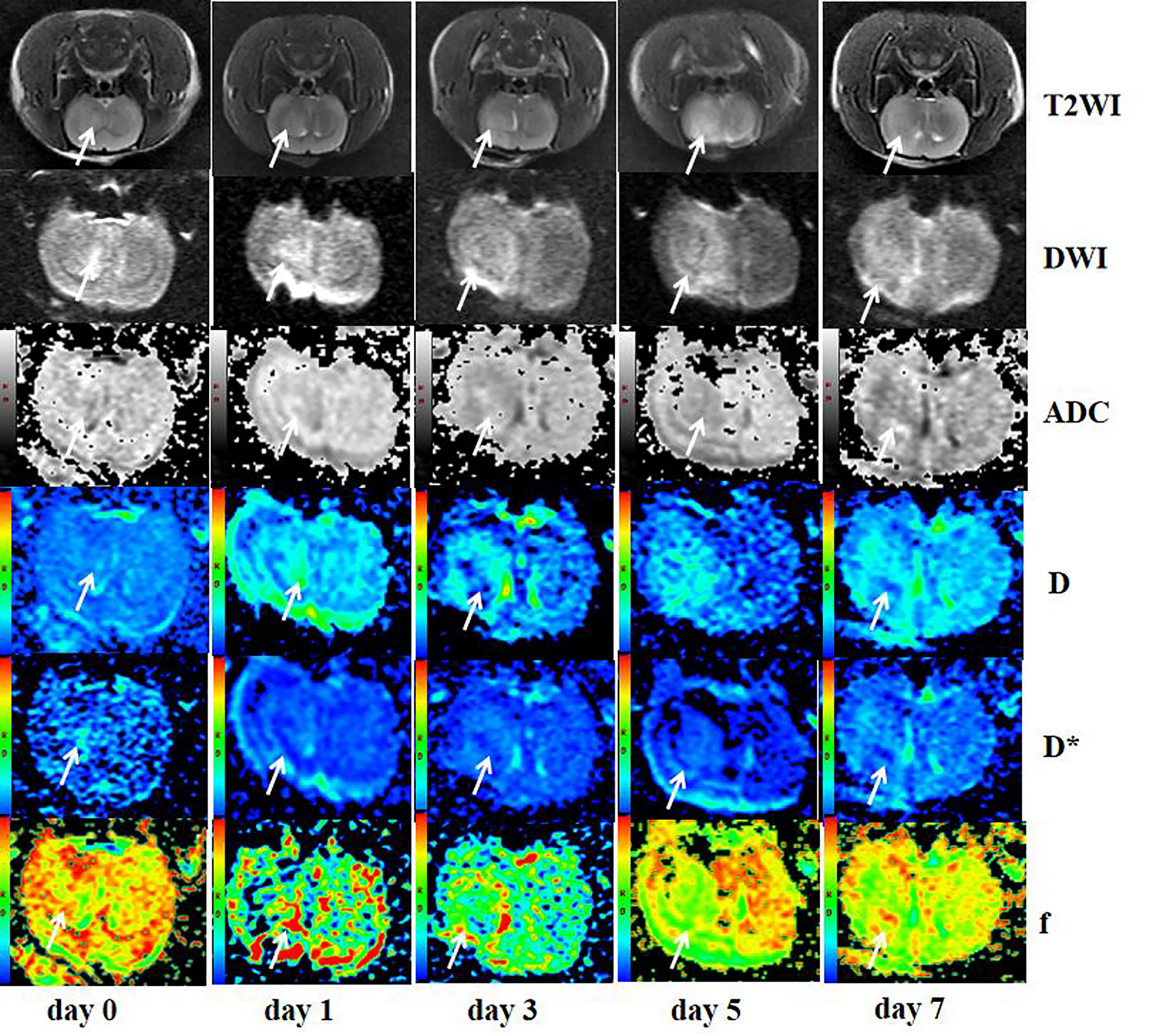
Axial T2WI, DWI (b = 1000), ADC, and pseudocolor maps of D, D*, and f at different time points in the treated group. T2WI image showed the tumor grew slowly from day 0 to day 7 (arrows) and presented hyperintensity on DWI images and hypointensity on ADC maps (arrows). Color ranging from blue to red represented values ranging from low to high. D-values increased gradually (arrows), while D*- and f-values decreased during the 7 days (arrows).

The gliomas were generally located in the right cerebral hemisphere with scattered hemorrhage, and tumors, as visualized on T2WI and IVIM-DWI (b = 1,000 s/mm^2^) images, were verified withs HE staining (**Figure 3**). T2WI image showed that tumors grew slowly from days 0 to day 7 in the treatment group and had indicative hyperintensities on DWI images and hypointensities on ADC maps, with colors, ranging from blue to red, representing values ranging from low to high. Pseudocolor maps indicated that the D-values increased gradually while D*- and f-values decreased during the 7 days (**Figure 4**).

### Intraobserver and Interobserver Agreement of IVIM-DWI Parameters

The reproducibility of ADC, D, and D* ranged from good to excellent and the reproducibility of f ranged from moderate to good. The interclass correlation coefficient of the IVIM-DWI parameters is summarized in **Table 1**.

**Table 1:** The interobserver and intraobserver reproducibility of IVIM-DWI parameters.

	Interobserver	Intraobserver
ADC	0.944 (0.702–0.956)	0.903 (0.783–0.936)
D	0.778 (0.692–0.895)	0.895 (0.744–0.921)
D*	0.856 (0.603–0.881)	0.842 (0.689–0.911)
f	0.648 (0.497–0.785)	0.731 (0.558–0.802)

The 95% confidence intervals of interclass correlation coefficient (ICC) were in parentheses. ADC, apparent diffusion coefficient; D, diffusion coefficient; D*, pseudodiffusion coeffificient; f, perfusion fraction.

### IVIM-DWI Parameter Analyses

**Figure 5:**
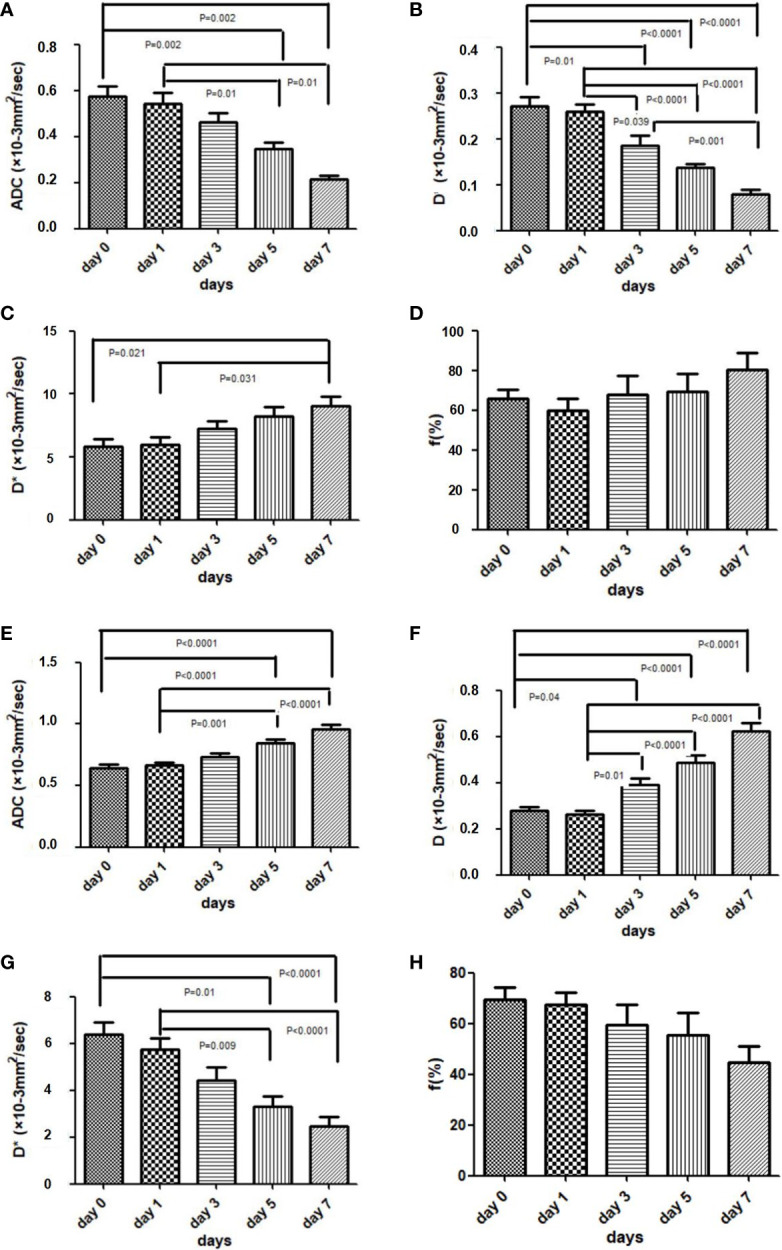
Box plots comparing the IVIM-DWI parameters in the control groups **(A–D)** and treated group **(E–H)** at different time points. The four IVIM-DWI parameters between the two groups all showed opposite increased or decreased trends. ANOVA followed by the Bonferroni test was used in multiple comparison of different time points. Error bars denote standard errors.

**Figure 6:**
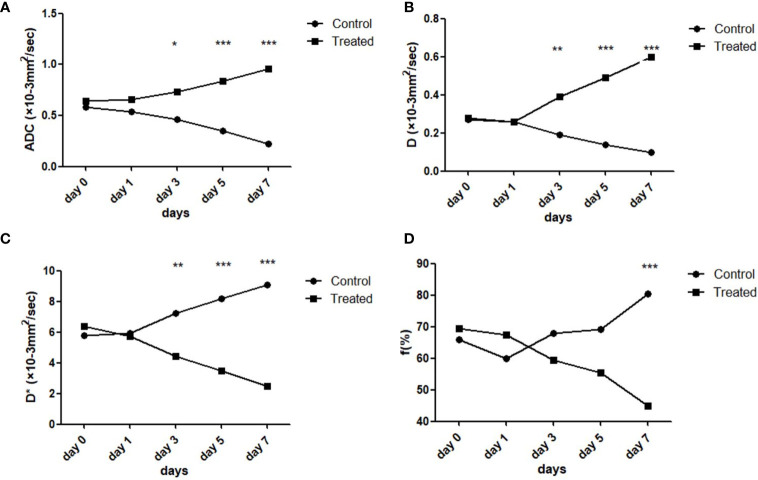
Line graphs comparing the IVIM-DWI parameters of the treated and control group on different time points. The four IVIM-DWI parameters between the two groups all showed opposite trends during therapy **(A–D)**. The independent-samples *t*-test was used. *p < 0.05, **p < 0.01, ***p < 0.001.

The dynamic changes of the IVIM-DWI parameters in the control and treatment groups at each time point are displayed in **Table 2**. ADC-value in the treatment group increased gradually while it decreased in the control group during the therapy course, with the two groups displaying significant differences among the five time points (treatment group: *F* = 18.72, *p* < 0.001; control group: *F* = 7.98, *p* < 0.001). Multiple comparisons between the two groups are shown in **Figures 5A, B**. For intergroup analyses, the baseline ADC-values were 0.585 ± 0.112 ×10^−3^ mm^2^/s and 0.643 ± 0.290 × 10^−3^ mm^2^/s in the control and treatment groups, respectively, which displayed no significant difference. However, the ADC-values in the treatment group were significantly higher than those in the control group from day 3 onwards (day 3: *p* = 0.033; day 5: *p* < 0.001; day 7: *p* < 0.001, **Figure 6A**).

**Table 2:** Comparison of IVIM-DWI parameters between the control and treated groups at different time points before and after antiangiogenic therapy.

	Day 0	Day 1	Day 3	Day 5	Day 7
ADC (×10^−3^ mm^2^/s)					
Control	0.585 ± 0.112	0.577 ± 0.126	0.487 ± 0.130	0.450 ± 0.150	0.363 ± 0.140
Treated	0.643 ± 0.290	0.668 ± 0.271	0.733 ± 0.182	0.846 ± 0.278	0.968 ± 0.340
*p*	0.215	0.055	0.033	<0.001	<0.001
D (×10^−3^ mm^2^/s)					
Control	0.274 ± 0.052	0.268 ± 0.040	0.192 ± 0.087	0.145 ± 0.058	0.086 ± 0.039
Treated	0.288 ± 0.050	0.265 ± 0.064	0.396 ± 0.150	0.499 ± 0.016	0.630 ± 0.290
*p*	0.730	0.798	0.052	<0.001	<0.001
D* (×10^−3^ mm^2^/s)					
Control	5.797 ± 1.642	5.946 ± 1.620	7.233 ± 2.760	8.189 ± 2.830	9.061 ± 1.870
Treated	6.374 ± 1.730	5.758 ± 1.450	4.446 ± 1.709	3.475 ± 1.440	3.290 ± 1.102
*p*	0.505	0.810	0.005	<0.001	<0.001
f (%)					
Control	65.833 ± 11.502	59.837 ± 14.230	68.440 ± 22.003	69.164 ± 22.340	80.335 ± 28.037
Treated	69.525 ± 11.320	67.325 ± 12.530	59.355 ± 20.026	55.329 ± 24.703	44.858 ± 14.930
*p*	0.581	0.368	0.498	0.304	0.008

Comparisons of IVIM-DWI parameters between the control and treated groups at the same time point were performed by the independent-samples t-test. ADC, apparent diffusion coefficient; D, diffusion coefficient; D*, pseudodiffusion coeffificient; f, perfusion fraction.

The D-value in the treatment group increased daily while it significantly decreased in the control group. There was significant difference of D-value among the five time points in both treatment and control groups (treatment: *F* = 33.17, *p* < 0.001; control group: *F* = 23.64, *p* < 0.001). Comparison results between the two groups are shown in **Figures 5C, D**. For intergroup analyses, the baseline D-values were 0.274 ± 0.052 × 10^−3^ mm^2^/s and 0.288 ± 0.050 ×10^−3^ mm^2^/s in the control and treatment groups, respectively, which displayed no significant difference. Following daily treatment of Bev, the D-values in the treatment group were significantly higher compared with those in the control group on days 5 and day 7 (both *p* < 0.001, **Figure 6B**).

For the perfusion-related parameters, the D*-value of the treatment group decreased gradually and displayed significant differences among the five time points (*F* = 11.08, *p* < 0.001). By contrast, the D*-value of the control group increased markedly with time (*F* = 4.23, *p* = 0.008). The results of multiple comparisons between the two groups are shown in **Figures 5E, F**. After treatment, D*-values in the treatment group were significantly lower than those in the control group on days 3, 5, and 7 (day 3: *p* = 0.005; day 5: *p* < 0.001; day 7: *p* < 0.001, **Figure 6C**).

The f-value of the treatment group decreased slightly during the treatment course while that of the control group increased during the 7 days; however, there was no difference among the five time points in the two groups (treatment group: *F* = 0.95, *p* = 0.45; control group: *F* = 2.08, *p* = 0.11, **Figures 5G, H**). Only the f-value on the 7th day showed a significant difference between the two groups (*p* = 0.008, **Figure 6D**).

### Correlation Between ΔVolume and IVIM Parameters

**Figure 7:**
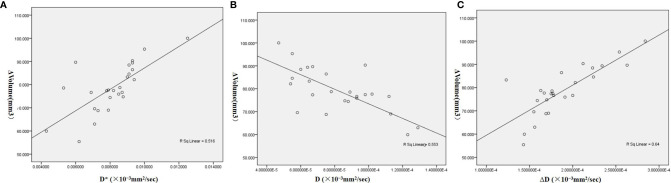
Scatter diagrams of correlations between the relative change of tumor size (ΔVolume) and IVIM-DWI parameters. **(A)** The initial D-value showed moderate negative correlation with ΔVolume. **(B, C)** Initial D*-value and relative change of D-value (ΔD) demonstrated moderate positive correlation with ΔVolume. The Pearson correlation analysis was used.

The initial D-value showed a moderate negative correlation with ΔVolume (γ = −0.744, *p* < 0.001), while the initial D*-value and relative change of D-value (ΔD) demonstrated moderate positive correlations with ΔVolume (D*: γ = 0.718, *p* < 0.001; ΔD: γ = 0.800, *p* < 0.001, **Figure 7**). No correlation between other initial or relative changes of IVIM parameters and tumor size were found.

### Histological Staining and Correlations With IVIM-DWI Parameters

**Figure 8:**
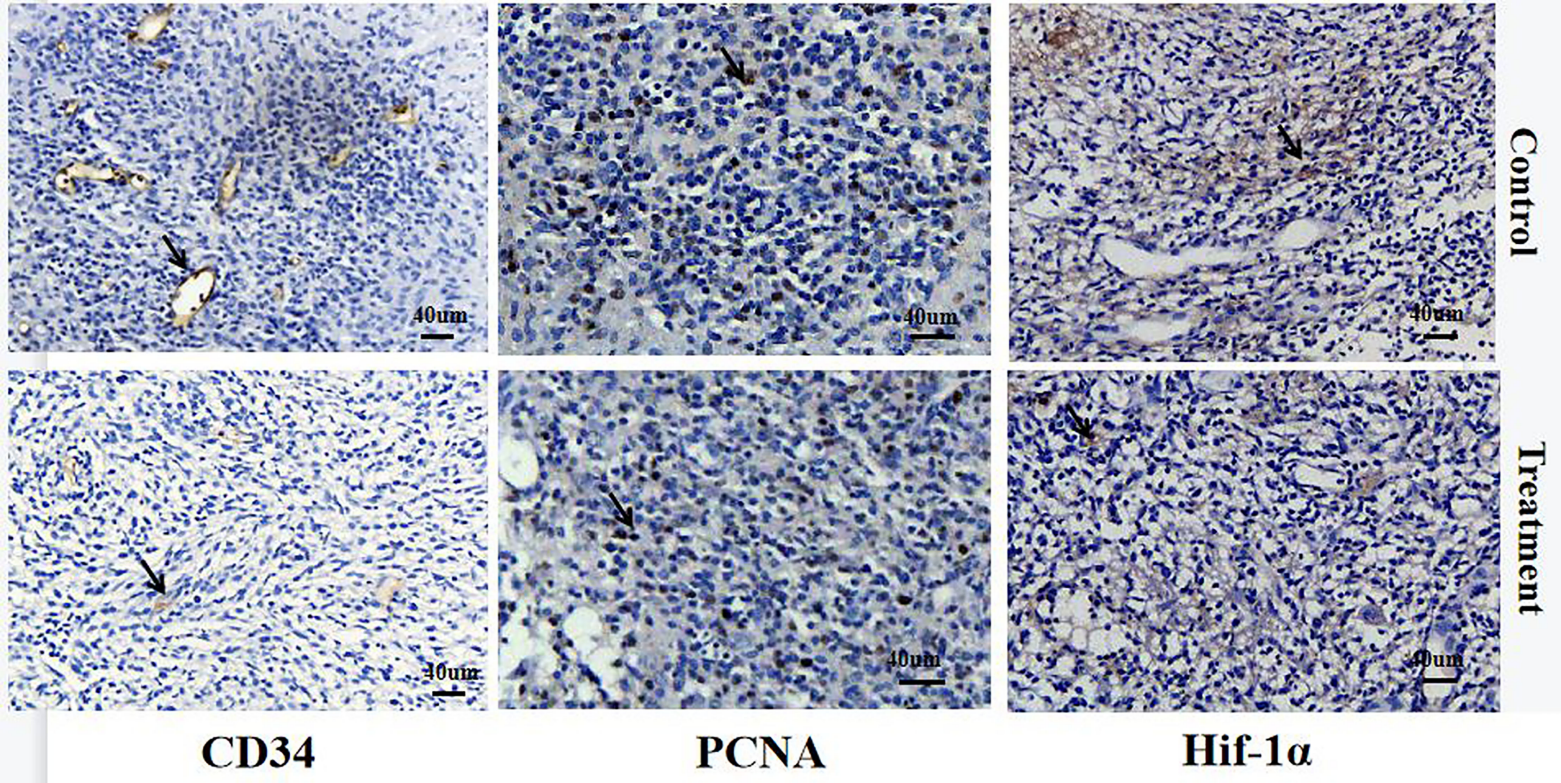
IHC staining of CD34, PCNA, and Hif-1α in the control and treated group on day 7 after therapy. Positive cells are indicated with arrows. The CD34-positive vessels in the control group were dilated and distorted while they were tiny and regular in the treatment group. The PCNA-positive cells in the treatment group was lower than that in the control group. The Hif-1α-positive area in the treatment group was smaller than that of the control group.

CD34. Vascular endothelial cells were stained yellow and brown in CD34-stained sections. The CD34-positive vessels in the control group were dilated and distorted while they were tiny and regular in the treatment group, with a significant difference in the MVD between the two groups on day 7 (*p* < 0.001, **Table 3** and **Figure 8**). In addition, MVD was strongly correlated with D*-value (*r* = 0.886, *p* = 0.019, **Table 4**).

**Table 3:** The scores and statistical differences analysis of MVD, PCNA, and Hif1-α between the control and treated groups on day 7 after antiangiogenic therapy.

Groups	MVD	PCNA	Hif1-α
Control	38.890 ± 6.571	10.896 ± 2.326	5.677 ± 1.213
Treated	18.328 ± 4.256	4.875 ± 1.191	2.940 ± 0.046
*p*	<0.001	<0.001	<0.001

Comparisons of histological assessment between the control and treated groups on day 7 were performed using the independent-samples t-test.

**Table 4:** Correlation coefficients between IVIM-DWI and immunohistochemistry scores of the treated group on day 7 after antiangiogenic therapy.

	MVD		PCNA		Hif-1α	
	Pearson’s *r*	*p*	Spearman *r*	*p*	Spearman *r*	*p*
ADC	0.767	0.075	−0.848	0.033*	−0.278	0.594
D	0.527	0.283	−0.928	0.008*	−0.879	0.010*
D*	0.886	0.019*	0.866	0.019*	−0.626	0.052
f	0.802	0.055	−0.314	0.544	−0.463	0.355

Pearson correlation was used for the comparison between MVD and IVIM-DWI parameters; Spearman’s analysis was used for comparisons between PCNA, Hif-1α, and IVIM DWI parameters (^*^p < 0.05).

Regarding PCNA, the cell nuclei were stained brown in PCNA-stained sections. The score of PCNA-positive cells in the treatment group was markedly lower than that in the control group on day 7 (*p* < 0.001, **Table 2** and **Figure 8**). PCNA was strongly correlated with ADC-value and the D-value (ADC: *r* = −0.848, *p* = 0.033; D: *r* = −0.928, *p* = 0.008, **Table 4**).

Regarding the Hif-1α, both the nuclei and cytoplasm of cells were stained brown in Hif-1α-stained sections. Hif-1α-positive cells were mainly distributed at the edge of the tumor necrosis area. The Hif-1α-positive area in the treatment group was markedly smaller than that of the control group, and showed a significant difference (*p* < 0.001, **Table 2** and **Figure 8**). The score of Hif-1α was strongly correlated with D-value (r = −0.879, P = 0.010, **Table 4**).

## Discussion

Based on our previous study, the orthotopic C6 glioma model exhibited greater growth rate with less necrosis or hemorrhage area from day 14 to day 21 after C6 cell transplantation (12). Therefore, we started anti-angiogenesis therapy from the 14th day after establishing the glioma model and continued the therapy for 7 days to obtain the early dynamic changes in IVIM-DWI parameters.

Based on the dual-exponential model of IVIM-DWI, the perfusion-related parameters f and D* are influenced by the blood volume and velocity of the tumor (13). In our study, the f and D*-values demonstrated a pronounced reduction in the treatment group, which proved the effect of Bev-mediated inhibition of VEGF-dependent vascular growth. Besides, the positive correlation between the initial D* and ΔVolume indicated that tumors with high blood perfusion responded better to anti-angiogenic therapy. This result was essentially in agreement with previous studies that the D* and f-values of malignant tumors decreased significantly after anti-angiogenic therapy (14–16).

Our previous study of DCE-MRI data from the orthotopic C6 glioma model showed slightly increased K^trans^ and K_ep_ on day 1 after anti-angiogenic therapy, which may be the result of decreased leakage and increased circulation presented in the “normalization of tumor vasculature” theory (17, 18). However, the transient increase of perfusion-related MRI parameters was not found in the current IVIM-DWI research, suggesting that there was no direct correlation between IVIM-DWI perfusion-related parameters and DCE-MRI perfusion-related parameters. In fact, previous research has indicated that the perfusion parameters of DCE-MRI and IVIM-DWI were not completely consistent and possibly even contrasting. Indeed, IVIM-DWI provides information on the total blood transit through a voxel and is focused on the intravascular blood movement, while DCE-MRI provides information on the exchange of contrast agents between the intravascular and extravascular spaces (19, 20).

CD34-positive tubules are a pivotal feature of microvascular growth and were used to calculate the density of neovascularization in our study. Our study showed that D*-value had a significant positive correlation with MVD, which indicated that the perfusion-related IVIM parameters could reflect the change of blood flow accurately in glioma models following intervention of anti-angiogenic therapy. Besides, although previous research found that the f-value had more potential to predict the tumor response after antiangiogenic therapy, there was no explicit correlation between MVD and f in our present study, a result that was likely because of the obvious image noise of the f pseudo-color image (21).

The diffusion-related parameters ADC and D reflect the microscopic Brownian motion of water molecules in tumor tissue (22). The decreased tumor blood perfusion following Bev treatment resulted in a decrease in cell proliferation, and resulted in a significant increase in ADC and D-values from day 5. Together, the data indicated that the change of diffusion-related parameters occurred later than that of perfusion-related parameters. The degree of proliferation can be reflected by PCNA because it is strongly associated with DNA replication in tumor cells (23). The decrease of tumor cells after anti-angiogenic therapy was also confirmed by the significant differences in PCNA scores between the control and treatment groups on day 7. The negative correlation between diffusion-related parameters and PCNA in the treatment group indicated that ADC and D-values could accurately reflect the cell density after anti-angiogenic therapy. The correlation between ADC-value and PCNA was weaker than that between D-value and PCNA, probably because the ADC-value was influenced by both microscopic water diffusion and blood perfusion of the tumor.

Hypoxia reduces the responsiveness of tumors to conventional chemotherapy and radiotherapy, and is an important factor of anti-angiogenic therapy failure for high-grade glioma (24). Previous research has confirmed that hypoxia is related to distorted neovascularization and proliferative cell density of tumors (25). In our study, we used Hif-1α as the pathological gold standard to evaluate the hypoxia of high-grade glioma and found that there was positive correlation between D-value and Hif-1α. The increased D-value indicated the reduced tumor cell density, which resulted in the decreased oxygen consumption of tumor cells. Our results confirmed that the diffusion-related parameter D-value could reflect the hypoxia of glioma after anti-angiogenic therapy. Unfortunately, we did not find any correlation between perfusion-related parameters of IVIM-DWI and Hif-1α, which may be related to the complex changes in blood vessels after anti-angiogenic therapy.

There were some limitations in the study. First, because of the limited sample size, the correlation between IVIM-DWI parameters and histological assessment was only analyzed at the end of follow-up. Second, combined therapy with temozolomide was not used in this study because we aimed to explore the correlation between IVIM-DWI parameters and immunohistochemical indicators, rather than to confirm the mechanism of anti-angiogenic medicines. Thus, future studies should focus on investigating the effects of anti-angiogenic therapy combined with traditional chemotherapy in the same model using IVIM-DWI.

## Conclusions

In conclusion, IVIM-DWI showed decreased perfusion-related parameters and increased diffusion-related parameters were consistent with histological staining in the Bev-treated glioma model. The present study demonstrated that IVIM-DWI was sensitive and accurate in predicting and monitoring the effect of early anti-angiogenic therapy in the C6 glioma rat model.

## Data Availability Statement

The raw data supporting the conclusions of this article will be made available by the authors, without undue reservation.

## Ethics Statement

The animal study was approved by the Ethics Committee for Animal Experimentation of Anhui Medical University of Anhui Province, China (Approval no. SCXK-Wan-2017–001) and was conducted in strict accordance with the Guidelines of the National Institutes of Health for the Care and Use of Laboratory Animals.

## Author Contributions

WH and YX contributed to conceptualization. YX, HP, and MX contributed to the cell and animal experiments. YQ, WH, and HP contributed to MRI scan and MRI data analysis. YS contributed to pathological analysis. WH contributed to writing the original draft. XL and YY contributed to writing, reviewing, and editing. All authors contributed to the article and approved the submitted version.

## Funding

This work was supported by the Natural Science Foundation of Anhui Province [No. 2008085QH381].

## Conflict of Interest

The authors declare that the research was conducted in the absence of any commercial or financial relationships that could be construed as a potential conflict of interest.

## Publisher’s Note

All claims expressed in this article are solely those of the authors and do not necessarily represent those of their affiliated organizations, or those of the publisher, the editors and the reviewers. Any product that may be evaluated in this article, or claim that may be made by its manufacturer, is not guaranteed or endorsed by the publisher.
